# Heritability of plumage colour morph variation in a wild population of promiscuous, long-lived Australian magpies

**DOI:** 10.1038/s41437-019-0212-4

**Published:** 2019-03-25

**Authors:** Ana E. Dobson, Daniel J. Schmidt, Jane M. Hughes

**Affiliations:** Australian Rivers Institute, Griffith University, Nathan Campus, Nathan, QLD 4111 Australia

**Keywords:** Quantitative trait, Genetic variation, Heritable quantitative trait, Quantitative trait, Genetic variation

## Abstract

Colour polymorphisms have evolutionary significance for the generation and maintenance of species diversity. Demonstrating heritability of polymorphic traits can be challenging for wild populations of long-lived species because accurate information is required on trait expression and familial relationships. The Australian magpie *Cracticus tibicen* has a continent-wide distribution featuring several distinct plumage morphs, differing primarily in colour of back feathers. Black or white-backed morphs occur in eastern Australia, with intermediate morphs common in a narrow hybrid zone where the two morphs meet. This study investigated heritability of back colour phenotypes in a hybrid zone population (Seymour, Victoria) based on long-term observational data and DNA samples collected over an 18 year period (1993–2010). High extra-pair paternity (~ 36% offspring), necessitated verification of parent–offspring relationships by parentage analysis. A total of 538 birds (221 parents and 317 offspring) from 36 territories were analysed. Back colour was a continuous trait scored on a five-morph scale in the field (0–4). High and consistent estimates of back colour heritability (*h*^2^) were obtained via weighted mid-parent regression (*h*^2^ = 0.94) and by animal models (*h*^2^ = 0.92, C.I. 0.80–0.99). Single-parent heritability estimates indicated neither maternal nor paternal non-genetic effects (e.g., parent body condition) played a large role in determining offspring back colour, and environmental effects of territory group and cohort contributed little to trait heritability. Distinctive back colouration of the Australian magpie behaves as a quantitative trait that is likely polygenic, although mechanisms responsible for maintaining these geographically structured morphs and the hybrid zone where they meet are unknown.

## Introduction

Many species exhibit some form of morphological polymorphism, and colour polymorphic species in particular offer biologists tractable systems to study a variety of evolutionary processes (Hugall and Stuart-Fox 2012; Jones et al. 2018; Roulin 2004; Svensson 2017). Birds are particularly polymorphic: approximately 3.5% of extant bird species exhibit some form of intraspecific plumage variation, and phylogenetic analyses indicate colour polymorphism seems to have evolved repeatedly throughout the history and radiation of avian taxa (Galeotti et al. 2003).

Neutral mechanisms suggested to at least partially explain colour polymorphisms include past historical processes such as bottlenecks and barriers to gene flow, allopatric isolation with or without secondary contact, as well as a balance between mutation and genetic drift (Cook 1992; Hoffman et al. 2006; Kimura 1984; Marshall and Ritland 2002; Roulin 2004). Genetic processes may also play a role, via genetic hitchhiking of pigmentation genes or even pleiotropic effects of other genes linked to fitness (Barton 2000). Selective mechanisms often invoked in investigations of such polymorphisms include apostatic selection, sexual selection and disruptive selection/ local adaptation (Galeotti et al. 2003).

In birds, plumage colours are a result of pigmentation, refraction of light by structural arrangements within feathers, or a combination of both pigment and structure. Melanins account for a great deal of colouration in Aves, and are responsible for most black/brown/red-brown/ and grey colour variation (Fox 1976). Production of melanins in animals is thought to be predominantly genetically determined and highly heritable (Buckley 1987; Hearing and Tsukamoto 1991; Majerus 1998; Roulin and Ducrest 2013), and these pigments can be expressed in the feathers, skin, beaks and claws of birds. White pigmentation in birds is most commonly a product of a lack of pigment and/or feather structure, and white feathers are generally weaker and more vulnerable to bacterial and pathogenic infection than pigmented feathers (Bonser 1995; Burtt 1986; Burtt Jr. and Ichida 2004; Mackintosh 2001), but not necessarily lice infestation (Bush et al. 2006).

An interesting case of intraspecific plumage variation is found in the Australian magpie (*Cracticus tibicen*), a medium sized passerine with a lifespan of 25 years, that lives in permanent territorial groups (Kaplan 2004). Although eight sub-species are currently recognised based on small differences in morphological characters (e.g., wingspan) (Schodde and Mason 1999), three distinct plumage forms are found across the Australian continent. Of these, two are referred to as white backed, and one black backed, with all forms displaying sexual dimorphism (Schodde and Mason 1999).

**Fig. 1:**
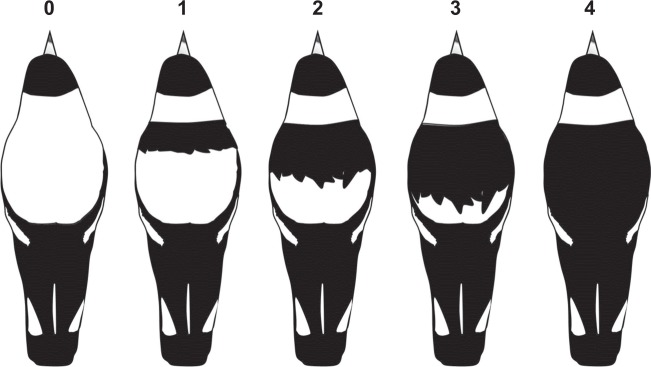
Australian magpie back colour variation. Schematic view of dorsal plumage showing five-morph scale (males only illustrated; females at the Seymour study site have grey, rather than completely white backs and napes)

Black backed forms that inhabit north-eastern Australia have white napes, shoulders and rumps, and a white tail terminated by a black band. All other feathered body parts, including the back, are black (see morph 4, Fig. 1). In south-eastern white-backed forms, the back is white instead of black, joining the white nape to the white rump and giving an appearance of an entirely white dorsal surface when bird is flying and viewed from above (morph 0, Fig. 1). Where distributions of the different plumage forms overlap, interbreeding is unrestricted (Burton and Martin 1976; Hughes 1982; Hughes et al. 2011), producing a range of different intermediate back colour forms (see morphs 1, 2, 3; Fig. 1). The hybrid zone in eastern Australia where white backs and black backs meet and intermediate forms occur is estimated to be 200 km wide (Burton and Martin 1976).

Genetic inheritance of back colour has been proposed based on theory and anecdotal observation, but is not yet supported by empirical data (Hughes 1982; Hughes et al. 2001). However it is clear that back colour pattern does not change within the lifetime of individual birds and the morphological pattern used to score this polymorphism (Fig. 1) is present in newly fledged chicks, juveniles and adults (Supplementary File 1). Observational data rule out plastic, seasonal or dietary back-colour variation in a long-term study site in the eastern Australian hybrid zone (Hughes et al. 2011). Analysis of variation in the melanocortin-1 receptor (MC1R) gene, a candidate gene for plumage colour differentiation in many taxa, found no association with back colour phenotype of Australian magpies (Dobson et al. 2012).

In southeast Australia, territorial groups range in size from 2–15 individuals and may defend a permanent habitat patch over many years (Hughes et al. 1996; Hughes et al. 2011). Rates of extra-group paternity (i.e., offspring fathered by males from outside the natal territory) found in a number of magpie populations are high in comparison to the majority of passerine species (Hughes et al. 2003). The level of extra-group paternity also seems to be highly variable between different populations, ranging from 26–88% (Durrant and Hughes 2005; Hughes et al. 2011).

Extra-pair and extra-group paternity (EPP and EGP) can lead to significant underestimations of the heritability of a trait of interest; and when these EPP/EGP are high (e.g., >25%), heritability estimates can be off by up to 40% (Bourret and Garant 2017; Charmantier and Reale 2005). The heritability of melanic plumage traits is generally relatively high (Roulin and Ducrest 2013), and the magpie is known to have high-to-very-high levels of EPP/EGP (Durrant and Hughes 2005; Hughes et al. 2003; Hughes et al. 2011). Thus, social parentage-based methods are unlikely to yield accurate estimates of a melanic plumage trait, such as the back colour variation observed within this species and estimates of genetic parentage are required in order to ensure heritability estimates are robust.

Within one of the hybrid zones of *C. tibicen’s* distribution, where black-backed (BB) and white-backed (WB) sub-species intergrade into a full range of intermediate back-colour forms, a large population outside the town of Seymour in Victoria has been the subject of a long-term study. At this study site, a mix of BB, WB and intermediate plumage forms coexist and interbreed (Hughes et al. 2002). Magpies and their highly territorial group associations have been continuously monitored at this site since the early 1990s, including an extensive programme of leg colour-banding and DNA sample collection (Finn and Hughes 2001). A previous study by Hughes et al. (2011), estimated an extra-pair paternity rate of 36%, and extra-group paternity rate of 26% in this population.

This study investigates back-colour inheritance within a hybrid zone population of Australian magpies, where pedigree-like data inferred from long-term field monitoring of banded birds has been genetically verified. Our aim was to estimate the proportion of back colour variability determined by additive genetic factors (i.e., narrow-sense heritability), and examine whether paternal, maternal or shared environmental effects significantly influence the narrow-sense heritability of back colour.

## Methods

### Study site and field methods

The Seymour study site consisted of a north-south transect of approximately 14 km along a road in which magpie territories either side of the road have been the subject of long-term monitoring. Over the 24 years of this longitudinal study, more than 140 territories have been surveyed and observed, with well over 2000 birds banded and DNA-sampled. Field methods presented here vary little from those outlined in Hughes et al. (2011). These territories were not static, but have formed, disappeared, shifted borders and composition over the years, so that at a given point in time there were generally no more than 90 territories under observation and/or in existence. During each fledging season, birds in each active territory were trapped, colour and number banded, and a DNA sample taken by toenail clipping toe IV (outer) and collecting 1–3 drops of blood into lysis buffer solution.

Colour banding enabled subsequent confident identification of individual birds at a distance, using telescopes and binoculars. Back colours were recorded at time of banding, as well as each time an individual’s back-colour was confidently observed during subsequent monitoring of territory occupancy. The assignment of back colour was based on a five-morph (0–4) scale reflecting the relative proportion of dark anterior vs. light posterior coloured sections on the back (0 being completely white-backed; 4 being completely black backed; see Fig. 1 and Supplementary File 1), and each observer over the 24 years of the study was trained and supervised by the same researcher (JMH). These scores were most commonly integers, but not exclusively, especially in cases where back colour is asymmetrical, and thus effectively measured a continuous trait as semi-continuous. In each extant territory, the presence of all banded and unbanded birds, along with back colour scores, were recorded during 20 min watches that were repeated six to eight times annually. This enabled fairly confident delineation of territory membership at a given point in time.

The present study utilised observational data and DNA samples from 36 territories in the Seymour population, and the data represent all fledglings and adults caught, banded and/or observed within these territories for a time period over which there was reliable sighting data and the majority of adult birds banded and bled. This varied between 4 and 14 years between territories (mean = 8.97 years; SD = 3.27), during the period spanning 1993 to 2010.

Territories were selected based on several factors including: completeness of watching data (annual confirmation of territory membership required sighting an individual on three 20 min watches) and whether all adult birds present were banded and bled. Back-colour observations for the 538 individual birds were calculated as the mean of all recorded sightings of a bird, helping to make back colour a somewhat continuous trait again. The number of back-colour observations per individual varied between 1 and 42 (mean = 6.89), and fledglings dominated the lower end of that spectrum, as many dispersed from their natal territory or died within the first year of being banded.

### Genetic methods

Eight variable microsatellite loci isolated specifically for *C. tibicen* and previously used for parentage analyses in magpie populations were utilised (see Supplementary Table S1; Durrant and Hughes 2005; Durrant and Hughes 2006; Hughes et al. 2003).

Two different genotyping methods were used over the course of this project. Initially PCR fragments were analysed using polyacrylamide gels as described in Hughes et al. (2011). From 2009 onwards, PCR fragment lengths were resolved on an ABI 3130 Genetic Analyser with a commercial standard (GeneScan™ -500 LIZ™, Applied Biosystems). Genotypes were then scored using Genemapper v4.0 (Applied Biosystems). To ensure the two genotyping methods were consistent, two reference magpie samples were used in both genotyping systems in addition to a calibration set of ~ 50 individuals selected from a range of territories that were genotyped and scored using both methods. The scoring of alleles of all individuals, within each territory, was rechecked as a group to ensure small differences between conditions in different PCRs and runs/gels were not misinterpreted as true variation. Mismatches between potential parents and fledglings (based on field observation) were also rerun at least twice to improve accuracy.

### Genetic analyses

Microsatellite genotypes were tested for the presence of null alleles using MICRO-CHECKER software (Van Oosterhout et al. 2004) and exact tests were performed on each locus in GENEPOP (Raymond and Rousset 1995) to test for deviation from Hardy-Weinberg proportions. The paternity analysis software package CERVUS (Marshall et al. 1998) was then used to statistically evaluate parentage exclusion probabilities for each possible fledgling-parent trio or pair.

In CERVUS analyses, simulation parameters assumed 85% of candidate mothers and 60% of candidate fathers in the population were sampled, to account for known high levels of extra-group paternity in this population, and for unsampled birds in surrounding groups and flocks (Durrant and Hughes 2005; Hughes et al. 2003). Parentage assignment in CERVUS followed the approach in Hughes et al. (2011). A strict confidence level of 90% and a maximum of one mismatching locus between parent and offspring, were used to reduce the false-positive rate, at the expense of inflating the number of false negatives and consequent loss of data. Initially, within each social territory, all females and males older than 1 year of age that were present within the previous or following 2 years, were tested as potential genetic parents of fledglings born in that territory. However, if maternity or paternity could not be assigned due to low confidence, fledglings were tested against adults from the surrounding 10–20 territories. Parental assignment required confidence levels (C.L.) at or above 90%, and parent-fledgling trio or pairs mismatching at a maximum of one locus. Mismatches were rechecked exhaustively for scoring errors, especially in cases of one microsatellite repeat unit difference. In cases where more than one potential mother or father matched at an equal number of loci, parentage was only assigned if a putative parents’ LOD score was above the 90% confidence level and/or positive assignment of the other parent sex enabled discrimination between the most likely of these putative parents using trio LOD scores.

### Heritability analyses

#### Regressions

To estimate heritability of back colour in this population, several different regressions were undertaken to best utilise all identified genetic relationships. Parent–offspring regressions are often utilised for the estimation of the narrow-sense/additive heritability (*h*^2^) of a phenotype (Lynch and Walsh 1998). These are robust even under conditions of assortative mating, and single-parent–offspring regressions (measuring half the heritability) can be used to identify possible maternal or paternal effects (Falconer 1975). Excel software and the Real Statistics Resource Pack software (Release 4.3) (Zaiontz 2014) were used for simple linear mid-parent/mid-offspring regressions, calculated on families in which parent pairs had had more than one offspring together (broods). Mother/mid-offspring, father/mid-offspring, as well as lightest parent/mid-offspring and darkest parent/mid-offspring regressions were also calculated to examine maternal, paternal, and dominance effects.

As a large number of parent pairs had only one offspring, the same regressions were repeated, but on back-colours of individual offspring (as opposed to mid-offspring values) for all parent combinations above. All parent/mid-offspring heritability estimates were weighted with an ICC (intra-class correlation coefficient), using iterative reweighting procedures until *h*^2^ values converged, in order to account for variance in the number of offspring within different broods, which ranged from 2–9 (mean = 3.63) following Lynch and Walsh (1998).

A restricted subset of data, composed only of families in which paternity and maternity confidence levels (C.L.) were above 90%, and parent-fledgling trio or pairs did not mismatch at any loci, was also examined using the same set of regressions in order to assess confidence in, and congruence with the larger dataset. Results of these additional analyses are omitted here but available in Supplementary File Table S3.

#### Animal models

A mixed-effects model (animal model), was also used to analyse heritability of back-colour. Animal models cope well with unbalanced designs and missing data, a common feature of long-term studies of natural breeding populations, and can utilise all of the complex familial relationships common in the pedigrees of natural breeding populations, as well as enable inclusion and estimation of potential sources of environmental variance (e.g., cohort, social group, birth year) within the model. Animal models are also relatively robust to bias from inbreeding or assortative mating (Lynch and Walsh 1998; Kruuk 2004).

The R package MCMCglmm (Version 2.22.1) (Hadfield 2010), implemented in R (Version 3.2.3) (R Development Core Team 2013), was used to generate an additive genetic relationship matrix from the genetically verified pedigree data, and this was used to fit a series of animal models with back colour phenotypes, including and excluding the random effects of territory of birth (i.e., social group sharing a habitat patch) and/or cohort (i.e., year of birth). Weakly informative priors were used on random effects (inverse-Gamma distribution with *nu* = 0.002 and *V* = 1). Models were run for 100 million iterations (burnin: 1 x 10^5^, thin: 3000) to reduce autocorrelation and were evaluated for convergence, good sampling and adequate effective sample size (ESS). Variance component ESS in all models ranged from 32,000 to 33,000. The posterior distribution of the heritability and its upper and lower 95% credible intervals were calculated, along with analogous variance components attributable to the random effects. Deviance information criterion (DIC) was used to compare model fit and complexity and determine if the inclusion of either or both random effects was justified (Hadfield 2010; Wilson et al. 2010).

## Results

### Parentage analysis

The number of microsatellite alleles ranged from 4 to 24 per locus, while heterozygosity varied between loci, from 0.40 to 0.877 (Supplementary Table 2). One locus (Gt206b) differed significantly from Hardy-Weinberg proportions after sequential Bonferroni correction for multiple tests; however, this locus has been previously used in parentage analyses for this population and others of the Australian magpie without issue (Durrant and Hughes 2005; Durrant and Hughes 2006; Hughes et al. 2003; Hughes et al. 2011). The very-high levels of relatedness within several sampled magpie territories are likely to be responsible for deviation of Gt206b (A. Dobson, unpublished data). Hughes et al. (2011), utilising the same microsatellite loci on the same population, used a subset of unrelated individuals to confirm that these eight loci satisfied Hardy-Weinberg proportions, and thus Gt206b was retained for all analyses.

The first assigned parent had an exclusion probability of 0.9992 from eight loci combined, and where both parents were assigned, this rose slightly to 0.9999 (Supplementary Table S2). Two parentage datasets from the Seymour population were combined for analyses of back colour: the first dataset encompassed 124 offspring from 17 territories at Seymour (1995 to 2005), for which both parents had been assigned in Hughes et al. (2011). The second dataset was composed of 193 offspring from 19 additional Seymour territories (1993–2009) where both parents were assigned and incorporating families with at least one assigned parent (Table 1).

**Table 1:** Summary of magpie samples sizes used in complete dataset analyses of heritability; combines birds from the 17 territories sampled in Hughes et al. (2011) with the 19 territories sampled for this study

Sampling group	Number
Individual offspring	Offspring
With both parents assigned	238
With mother assigned	292
With father assigned	262
With only mother assigned	55
With only father assigned	24
Family groups (≥ 2 offspring)	Families
With both parents assigned	54
With mother assigned	66
With father assigned	58
With only mother assigned	12
With only father assigned	4

### Assortative mating and inheritance patterns

Back colour morph of female and male magpie pairs that produced fledglings sampled in this study were examined for evidence of assortative mating. The correlation value (0.12) between female and male back colours of these mating pairs was not statistically significant (*t* = 0.11, *p* = 0.92). The distribution of the back colour pairings reflect frequencies of back colours observed in the sampled population, with dark backed individuals occurring at a higher frequency than white backs at this location within the hybrid zone across a 24 year observation period (Jane Hughes, unpublished data; Supplementary File Fig. S2).

**Fig. 2:**
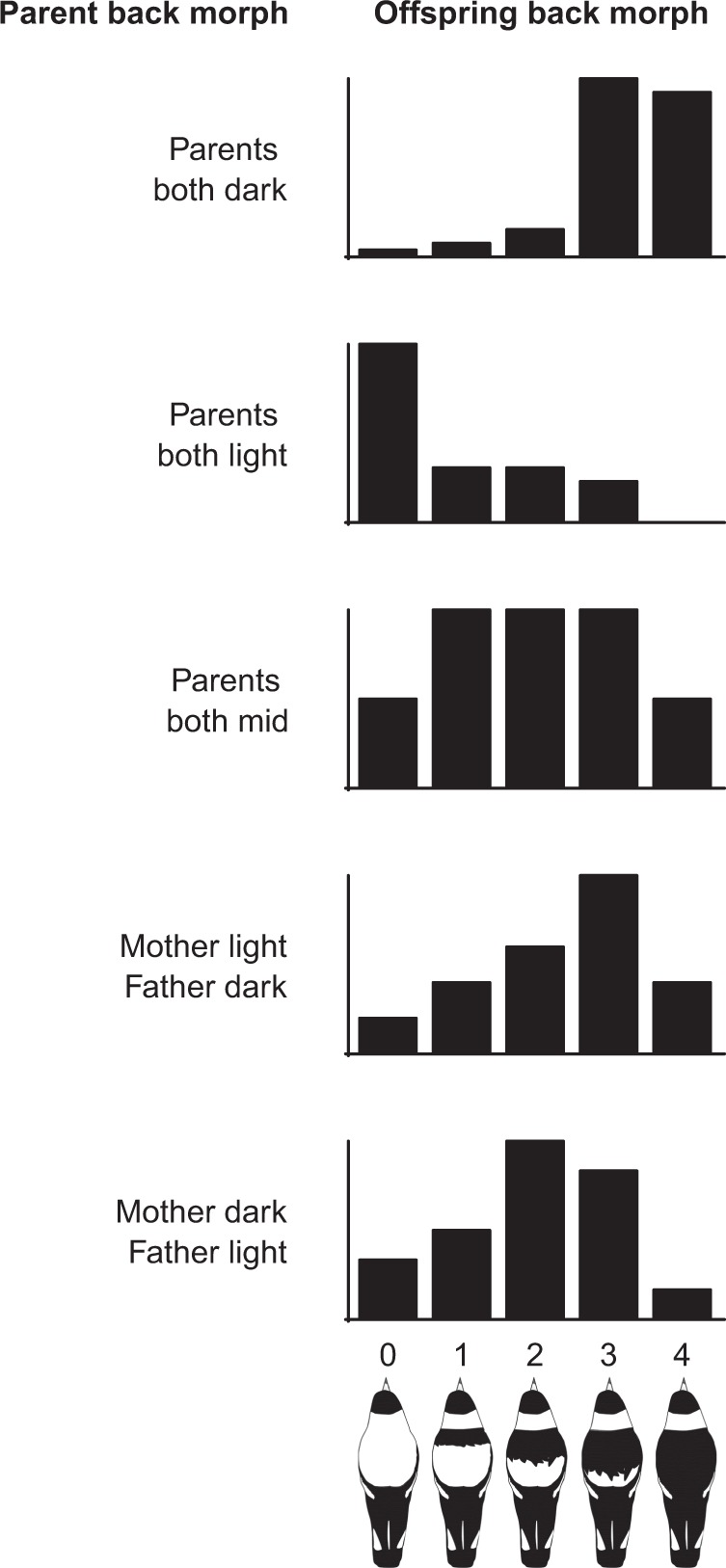
Offspring back morph histograms for different combinations of parental back colours. The parental designation ‘light’ includes bird with back colours from 0–1.5, ‘mid’ includes back colours 1.5–3, and ‘dark’ includes back colours 3–4

The range of offspring produced by different combinations of parental colours indicate a trend towards like-producing-like, as two ‘light’ backed parents (morph score 0–1.5) produced mostly light backed offspring, and two ‘dark’ backed parents (morph score 3–4) produced mostly dark backed offspring (Fig. 2). However opposite colour morph pairings, and pairs of mid back colour parents (morph score 1.5–3) produce an almost normal distribution of all back colours, centred near the most intermediate phenotype (Fig. 2).

### Regressions

Heritability estimates presented below reflect parentage assignment at a confidence level of ≥90%, and with ≤1 loci mismatch (an analysis of a subset of this data, comprised only of kin relationships confirmed under stricter criteria (C.L ≥90%, 0 loci mismatching) is included in Supplementary File Table S3).

**Fig. 3:**
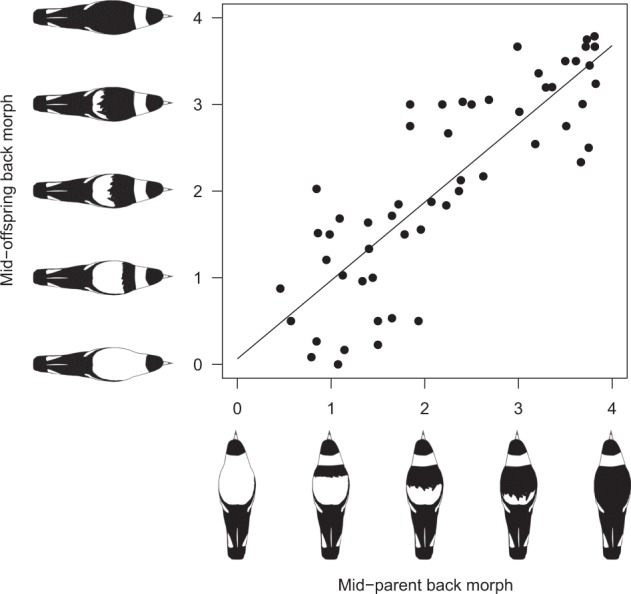
Regressions of mid-parent–mid-offspring back colour in a population of Australian magpies at Seymour, calculated on the complete dataset. Data are for 54 broods (brood size *μ* = 3.64, *σ* = 1.77) comprising 196 offspring and 86 parents, from 31 territorial groups during the period 1993–2009. *R*^2^ = 0.70; ICC-weighted *h*^2^ = 0.94

In all kin back colour regressions calculated, heritability estimates were highly significant (*p* ≤ 0.001) (Table 2 and Fig. 3). Regressions of individual offspring back colour, on one or both parents, indicated a range of *h*^2^ from 0.34 to 0.6 (Table 2). For families with multiple offspring, regressions of mid-offspring back colour upon single parents or parent pairs ranged from 0.48–1.0 (Table 2). Regressions of mid-offspring colour on the darkest and the lightest parent colour yielded heritability estimates of 0.48 and 0.61, respectively (Table 2).

**Table 2:** Kin regressions using complete dataset: heritability estimates for *C. tibicen* back-colour variability in the Seymour population

Back colour regression	*N*	*h* ^2^	SE	*p*-value	ICC-weighted *h*^2^	SE weighted
Father–individual offspring	262	0.600	0.06	6.60E-22		
Mother–individual offspring	292	0.340	0.05	2.00E-13		
Parent mid-point–individual offspring	238	0.441	0.06	1.27E-31		
Darkest parent–mid-offspring	54 (196)	0.480	0.11	4.82E-09		
Lightest parent–mid-offspring	54 (196)	0.617	0.07	2.05E-12		
Father–mid-offspring	58 (210)	0.530	0.08	1.18E-10	0.92	0.08
Mother–mid-offspring	66 (235)	0.540	0.08	7.84E-06	0.86	0.09
Parent mid-point–mid-offspring	54 (196)	0.702	0.08	5.77E-33	0.942	0.08

Values in brackets refer to total numbers of offspring within all families

Once weighted using intra-class correlation, the estimated heritability of back colour variation increased markedly and similarly across all regression types. Parent mid-point–offspring mid-point regression (corrected for unequal family size) is considered the most robust regression-based measure of narrow-sense heritability out of all the types of kin relationships calculated in this study (Lynch and Walsh 1998), and after ICC weighting, this was *h*^2^ = 0.94, indicating a high level of heritability of magpie back colour variation (Table 2 and Fig. 3).

Although heritability estimates obtained from mother–offspring regressions were lower than those obtained from father–offspring estimates, this difference almost disappears when regressions are weighted to account for differing family sizes. Back colour *h*^2^ based on corrected mother–offspring regression was estimated at 0.86, and *h*^2^ based on corrected father–offspring regressions estimated at 0.92 (Table 2), indicating this was likely to have been an artefact of the tendency of father–offspring ‘families’ to be larger; a consequence of the social structure of magpie territories in this population where territories often include a single dominant male and multiple breeding females (Hughes et al. 2011).

### Animal models

Heritability estimates of magpie back colour variation, obtained using animal models (*h*^2^ = 0.88–0.94) (Table 3) agreed with ICC-weighted regression estimates (*h*^2^ = 0.84–0.92) (Table 2). Inclusion of the different additional random effects of year of birth and territory of birth within animal models affected estimates of additive heritability in different ways (Table 3). Fitting the most complex model (model 4) reduced the *h*^2^ estimate and broadened its 95% confidence interval substantially, but increased the information criterion (DIC). Model 2 was found to be most parsimonious; the addition of the random factor of year of birth substantially improved model fit for magpie back colour, with the lowest DIC value by a large margin. Therefore, to avoid biased parameter estimation, model 2 was chosen to estimate heritability of this trait (*h*^2^ = 0.92.

**Table 3:** Generalised linear mixed models (GLMMs) (animal models) of magpie back colour with and without the random effect of cohort (i.e., year of birth; C) and territory of birth (T)

Random effect(s)	*V*_A_/*V*_P_ = *h*^2^	*V*_C_/*V*_P_	*V*_T_/*V*_P_	DIC
Model 1: ~ Back colour	0.91 (0.80–0.99)			382.51
Model 2: ~ Back colour + year[C]	0.92 (0.80–0.99)	0.008 (10^−4^–0.03)		317.65
Model 3: ~ Back colour + territory[T]	0.89 (0.75–0.99)		0.06 (10^−4^–0.18)	500.35
Model 4: ~ Back colour + year[C] + territory[T]	0.84 (0.65–0.99)	0.007 (7 × 10^−5^–0.03)	0.06 (8 × 10^−5^–0.18)	444.27

Heritability (*h*^2^) values include 95% credible intervals in parenthesis. *V*_A_ = additive genetic variance; *V*_P_ = phenotypic variance; *V*_C_ = cohort (year of birth) variance and *V*_T_ = territory variance. Analyses included data from 406 birds, including 288 offspring from 35 territories between 1993 and 2009. Some individuals were included as both offspring and parents

## Discussion

A review by Visscher et al. (2008) compared heritability values across a range of animal taxa and suggested that heritability is generally higher for morphological traits than for fitness traits (see also Merila and Sheldon 2000; Mousseau and Roff 1987). Of the traits reviewed, morphological trait heritability (*h*^2^) estimates ranged between 0.3 and 0.8 (*µ* = 0.43), while fitness traits ranged between 0.05 and 0.3 (*µ* = 0.18) (Visscher et al. 2008).

Within birds, heritability of colour trait variation seems to be strongly related to the type of pigmentation. Melanin-based traits generally have much higher estimated heritability than carotenoid or structurally based pigmentation, and melanic trait heritability estimates ranging between 0.8 and 0.95 are commonly estimated for bird taxa (see below). Therefore, the high heritability estimates for magpie back colour variation found in this study (weighted mid-parent regression: *h*^2^ = 0.94; animal model: *h*^2^ = 0.92, C.I. 0.80–0.99; Tables 2,3 and Fig. 3) seem likely to be an accurate reflection of a high genetic contribution to back colour variation in the study population. This conclusion is further supported by the fact that both traditional regression-based estimates and linear mixed model-based estimates produced congruent estimates of heritability. Importantly, high heritability estimates presented here are based on a genetic pedigree, avoiding bias associated with social pedigrees in species with extra-pair reproduction (Bourret and Garant 2017). Additionally, estimates are not inflated by assortative mating as no such effect was detected in agreement with previous studies (Hughes et al. 2011).

Maternal and paternal effects seem unlikely to have significantly influenced heritability estimates, as both maternal–offspring regressions and paternal–offspring back colour regressions estimated lower heritability values than mid-parent–offspring back colour regressions (Table 2 and Supplementary Fig. S1). The inclusion of shared environmental effects, in the form of patch quality (territory of birth) and cohort (year of birth), in the animal model indicated these may account for a very small proportion of the variance in this trait (see Table 3). However, measures of model fit indicated the inclusion of patch quality led to a lack of fit between models and data, as well as over-parameterisation of the model, and indicated the model with most support included the cohort effect only (Table 3). Generally, it is suggested that not accounting for shared environmental effects artificially inflates measures of heritability (Lynch and Walsh 1998). However, the inclusion of cohort as an effect resulted in a slightly higher *h*^2^ estimate (*h*^2^ = 0.92) than a model with no random effects (*h*^2^ = 0.91), indicating that not including the cohort term very slightly increased the error term of the model rather than artificially inflating the heritability estimate. If we accept the model with the lowest DIC, (Model 2: back colour and year of birth), the estimated *h*^2^ value of 0.92 (95% CI 0.8–0.99) is indeed highly congruent with *h*^2^ estimates obtained using weighted regressions of mid-parent on mid-offspring of 0.94.

Our analyses indicate a very-high level of back colour heritability in this population of Australian magpies—on par with melanin-based trait heritability estimates for other bird species. Several other points are consistent with this finding. First, magpie back colour has been observed to be stable at the level of individuals, both spatially and temporally, and does not materially change across seasons or moults. Second, melanin-based traits have often been shown to follow Mendelian laws of inheritance across several bird species, indicating these traits are under strong genetic control, rather than environmental or body condition-dependant (Roulin 2004). This makes sense in light of the fact that melanins are endogenously produced, in contrast to the majority of carotenoid-based plumage traits, which are heavily dependent upon environmental sources (Fox 1976; McGraw 2006; Roulin and Ducrest 2013). Third, a number of other bird species that express different morphs of melanic-based phenotypic traits have been found to have estimated heritability levels of a similarly high magnitude, including tawny owls (0.93) (Gasparini et al. 2009), barn owls (0.81) (Roulin and Dijkstra 2003), barn swallows (0.8) (Saino et al. 2013), alpine swifts (0.78) (Bize et al. 2006), pied flycatchers (0.31–0.93) (Potti and Canal 2011), common kestrels (0.67–0.83) (Kim et al. 2013) and common buzzards (0.82) (Kappers et al. 2018). These contrast dramatically with heritability estimates for some non-melanin-based variation where variation is often chromatic rather than polymorphic, such as the carotenoid chest (0.07) and structurallybased crown (0.10) in blue tits (Hadfield et al. 2006), and the carotenoid-based ventral patch in the great tit (0.29) (Evans and Sheldon 2012).

Although this study does not reveal information on the polygenic architecture of this trait, Hughes and Mather (1980) suggested a small number of genes may determine magpie back colour, and Hughes (1982) put forward a model based on two genes, with black back colour the dominant allele in both genes. They demonstrated how a polygenic system could explain the previously observed asymmetry in frequencies of back colours across the eastern hybrid zone (Burton and Martin 1976). As magpie back colour variation also has a sexually dimorphic component, it is plausible that this trait may have a component of sex-linked inheritance, and if so, animal model heritability estimates of autosomal additive variance presented here could potentially be inflated by the effect of sex-linked inheritance. Methods developed to model and test for sex-linked inheritance using modified animal models have been used in a number of bird species (Husby et al. 2012). Future studies of magpie back colour would benefit from partitioning heritability into autosomal and sex-linked components, which would require larger pedigrees and DNA-based sex identification for individuals observed only as juveniles.

The range of offspring back colours produced by different crosses of parental phenotypes in this study (Fig. 2) do show some support for a dominance of black back alleles, at least at one locus (if not more), as two black backed parents could produce a white-backed offspring, but two white-backed parents have yet to be found to produce completely black backed offspring (across 238 offspring born across 35 territories in a 16 year period). Incomplete dominance effects seem less likely, given intermediate colour forms are not true ‘mixes’ of ‘blends’ of black and white phenotypes (i.e., grey or similar), but express both white and black phenotypes over varying proportions of their backs, which itself is more suggestive of some type of co-dominance effects between phenotypes. It also remains quite possible that more complex mechanisms involving incomplete penetrance and/or differing levels of expressivity could potentially be involved in magpie back colour variation. The continuous nature of magpie back colour does, however, indicate that in the case of a hypothetical two-gene model of inheritance, at least one gene would likely need to be a modifier of some type i.e., control the width of the black band on the back, as was first suggested in Hughes (1982).

The low environmental contribution to magpie back colour variation implied by the high heritability estimates could be different in different populations or timeframes. This is because heritability can vary within and between populations, as well as temporally, and a pattern of higher heritability of morphological traits in ‘better’ or more stable environments may be a result of decreased environmental variance in such environments (Visscher et al. 2008). It would be interesting, but impractical, to compare heritability estimates for magpie back colour in poorer-quality habitats within a hybrid zone, even using alternative non-pedigree-based methods of estimating heritability (Ritland 1996; Thomas et al. 2002; Thomas and Hill 2000), and some studies indicate these methods may still be less likely to yield heritability estimates as accurate as those obtained using traditional pedigree-based methods (Thomas et al. 2002). Realistically, as heritability estimates in this population from data spanning several decades are very-high, it seems reasonable to suggest that heritability of back colour variation is also likely to be high in other polymorphic magpie populations across the range of the species (Visscher et al. 2008).

## Conclusions

Evidence presented here indicates that heritability of back colour variation is high in Australian magpies within the eastern hybrid zone, and that this high heritability is consistently estimated by two different analytical methods. This result is not unexpected for a melanic plumage trait, which generally show higher heritability than carotenoid or structurally based plumage colour traits. Single-parent heritability estimates indicate that neither maternal nor paternal non-genetic effects (e.g., parent body condition), play a large role in determining offspring back colour, and animal models indicate the environmental effects of patch quality and cohort also contribute very little to the heritability of this trait in magpies. Patterns of inheritance in familial groups suggest there may be some effect of dominance of black backed alleles, and that at least two genes are likely to play a role in determining magpie back colour. The high heritability of back colour variation found in this study also indicates that whole-genome sequencing may be profitable for further elucidation of the genetic basis of plumage colour variation in this species and others (e.g., Bourgeois et al. 2017; Toews et al. 2016).

### Data archiving

Data available from the Dryad Digital Repository: https://doi.org/10.5061/dryad.fc384rk

## Supplementary information

Supplementary File 1

Supplementary Tables and Figures
