# Supplementary material for: Heritability of plumage colour morph variation in a wild population of promiscuous, long-lived Australian magpies
Source: Heredity (Edinb). 2019 Mar 25;123(3):349–58. doi: 10.1038/s41437-019-0212-4 (PMC6781111; doi:10.1038/s41437-019-0212-4)

Supplementary File 1

Dobson et al. Heritability of plumage colour morph variation in a wild population of promiscuous, long-lived Australian magpies

Australian magpie back colour morphs – variation within individuals.

This document addresses the question of within-individual variation in the back colour morph trait. That is, whether back colour morph changes during the lifetime of an individual bird.

Each time an individual magpie was identified during a 20 minute watch period, it was assigned a back colour score based on a five-morph (0-4) scale reflecting the relative proportion of dark anterior versus light posterior coloured sections on the back (0 being completely white-backed; 4 being completely black-backed; see Fig. 1, 2).

Figure 1: Australian magpie schematic, dorsal perspective illustrating back colour morph scale, 0-4.

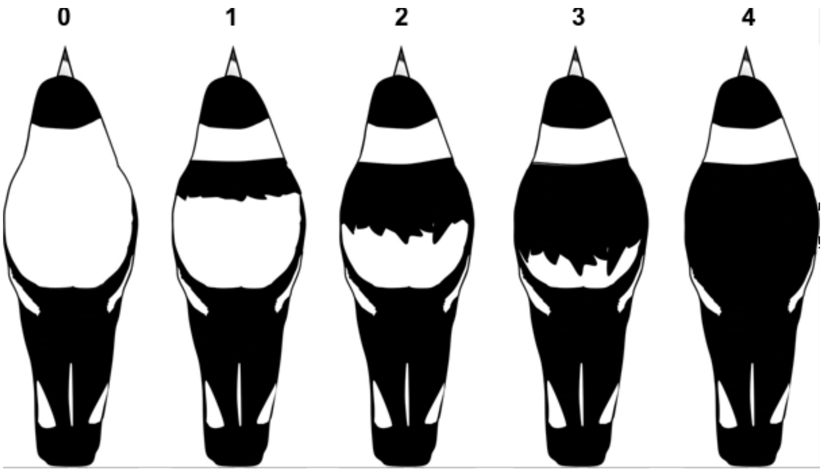

Figure 2: Images of Australian magpie adult males, illustrating back colour morph "0" (left), and back colour morph "4" (right).

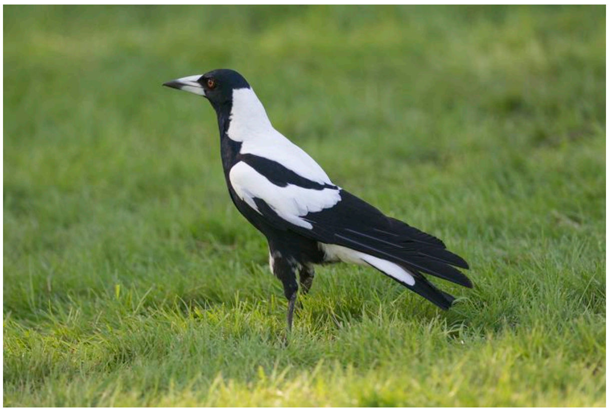

Australian Magpie, Fitzroy Gardens, Melbourne, Victoria.
Photographer: David Paul
Museums Victoria
https://collections.museumvictoria.com.au/species/8177

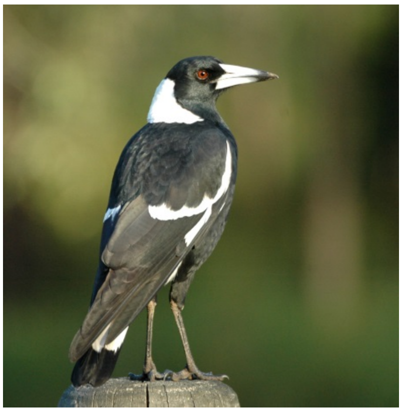

Australian magpie, Queensland
By Aviceda - Own work, CC BY-SA 3.0,
https://commons.wikimedia.org/w/index.php?curid=5528647

The 5 morph scale used in this study can be assessed in fledged chicks, juveniles and adults (adult plumage attained at 3 years) because it represents the relative proportion of dark (anterior) versus light (posterior) coloured sections on the back. In chicks these coloured sections appear as dark grey and light grey, changing to white and black as adult plumage is attained - but the relative area of the two sections is clear at all ages and it is the relative area of the dark and light sections that is the basis of the morph scale, not shading. We are not aware of any published or anecdotal reports of Australian magpie back colour morph changing during the life of an individual.

Some measurement error is expected when recording back colour morphs, as it's an observation made in the field by multiple observers in different weather conditions and from different distances. Every time a bird was observed, its back colour (five morph scale) was recorded by the observer. The coefficient of variation for repeated back colour observations on individual birds was 10.8% (n = 319), so multiple observations on the same bird over time (years) tend to be consistent. We attribute variation in individual back colour scores to measurement error, not ontogenetic change in plumage of birds. This is supported by examining whether back colour scores change over consecutive observations for birds observed over at least 10 watch periods (>3 years). The distribution of slopes for these relationships for 111 birds is centered on zero and not significantly different from zero ($t = 1.37$, $df = 110$, $p = 0.18$; see Fig. 3). So the small amount of variation in back colour scoring of individual birds shows no evidence for consistent change over time and is more likely due to measurement error. For this reason, the back colour morph score used as response variable in our analysis of heritability is taken as the mean of all values recorded during the study for that individual.

Figure 3:

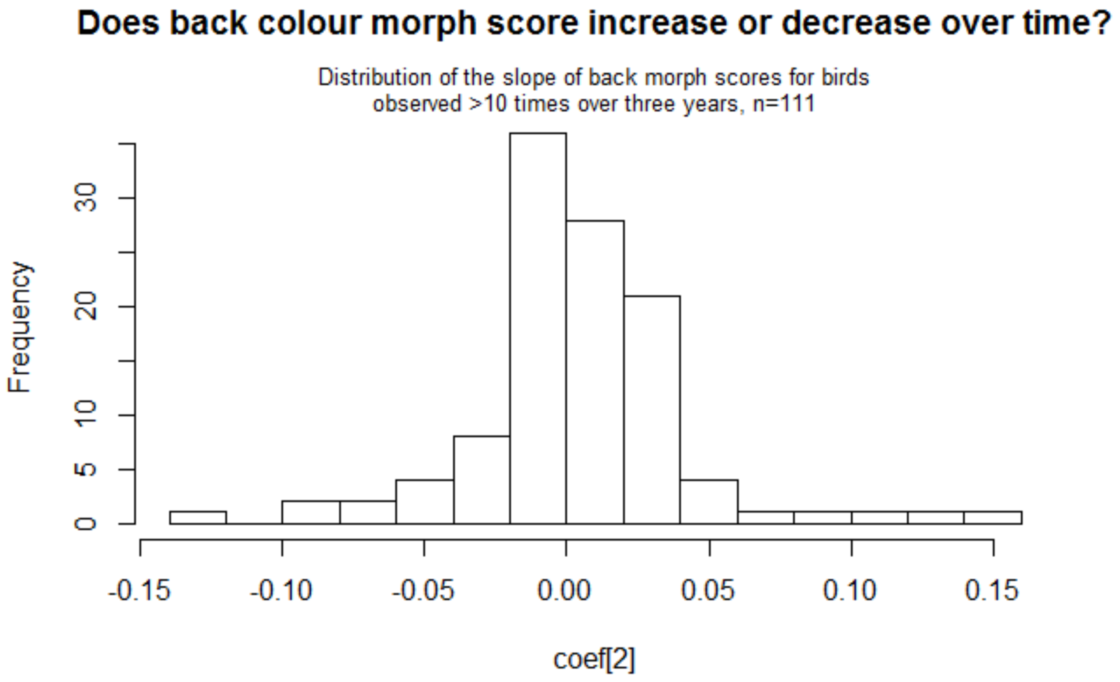

Supplement: Supplementary file 1 [file 41437_2019_212_MOESM1_ESM.pdf]
