# Supplementary material for: Heritability of plumage colour morph variation in a wild population of promiscuous, long-lived Australian magpies
Source: Heredity (Edinb). 2019 Mar 25;123(3):349–58. doi: 10.1038/s41437-019-0212-4 (PMC6781111; doi:10.1038/s41437-019-0212-4)
Supplement: Supplementary file 2 — Supplementary Tables and Figures [file 41437_2019_212_MOESM2_ESM.docx]

**Supplementary Table S1:** Primer sequences and annealing temperatures for eight microsatellite loci developed for parentage in the Australian magpie (Hughes et al., 2003, Durrant and Hughes, 2005).

**Supplementary Table S2:** Summary statistics of microsatellite loci used in parentage analysis.

**Supplementary Table S3:** Kin regressions using strict data subset (Cervus analysis C.L ≥90%, 0 loci mismatching): heritability estimates for *C. tibicen* back-colour variability in the Seymour population. Values in brackets refer to total numbers of offspring within all families.

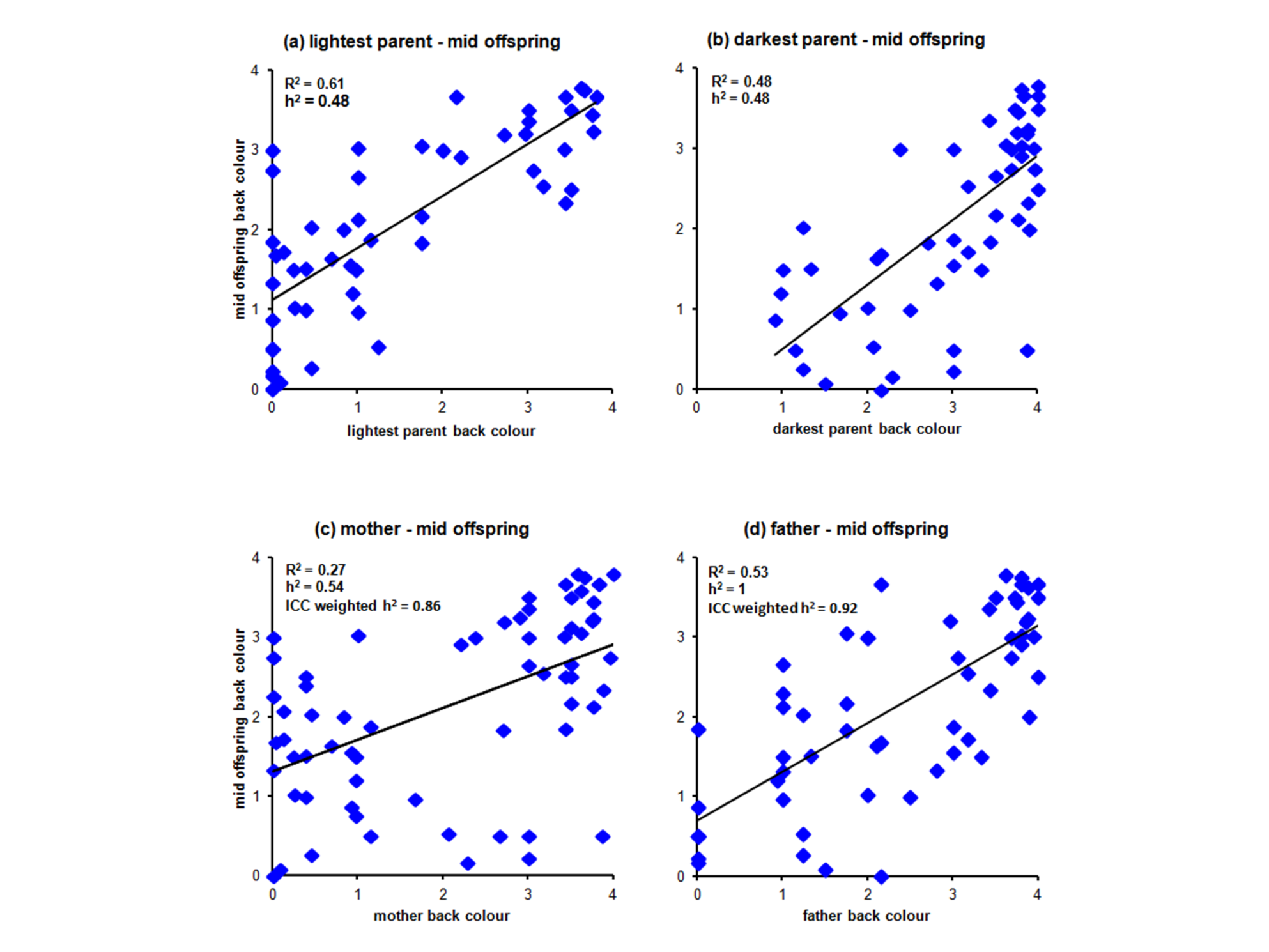


**Supplementary Figure S1:** Complete dataset regressions of parent-offspring back colour, in a population of Australian magpies at Seymour calculated based on the complete dataset. Birds were from 30-32 territorial groups during the period 1993-2009. (a-d) refer to individual regressions of mid offspring back colour onto different parents by sex and by back colour.

**Supplementary Figure S2:** Frequency distribution of magpie back colours in analysed territories of the Seymour study population.
